# Effect of Prestrain on Payne Effect and Hysteresis Loss of Carbon-Black-Filled Rubber Vulcanizates: Measurements and Modeling

**DOI:** 10.3390/polym16030436

**Published:** 2024-02-04

**Authors:** Boyuan Yin, Xinyue Jiao, Haibo Wen, Yan Li, Ming Li

**Affiliations:** 1 School of Civil Engineering, Hunan University of Science and Technology, Xiangtan 411201, China; 23020201011@mail.hnust.edu.cn (X.J.); 21020201085@mail.hnust.edu.cn (H.W.); 2 Department of Mechanical Engineering, Hunan Institute of Engineering, Xiangtan 411101, China; liyan304@hnie.edu.cn; 3 School of Civil Engineering and Architecture, Hunan University of Arts and Science, Changde 415000, China; liming@huas.edu.cn

**Keywords:** CB-filled rubber, prestrain, Payne effect, hysteresis loss

## Abstract

The performance of a viscoelastic damper is governed by the mechanical properties of the viscoelastic material, which are sensitive to prestrain. Among viscoelastic materials, carbon black (CB)-filled rubber vulcanizate is commonly used in structural applications. In this paper, the prestrain-dependent Payne effect and hysteresis loss of CB-filled rubber vulcanizates are investigated through experimental and theoretical analysis. Based on the experimental results, the classic quantitative models proposed by Kraus, Huber–Vilgis, and Maier–Göritz are used to describe the Payne effect. The results show that the Maier–Göritz model is most suitable to describe the Payne effect, especially for the loss modulus. After calculating the area of the hysteresis loops, hysteresis loss curves at various dynamic strain amplitudes are parallel to each other. Through application of the time–strain superposition principle, the hysteresis loss at any arbitrary prestrain can be predicted. Thus, the aim of this paper is to provide guidance for researchers in choosing an accurate model for future investigations of the prestrain-dependent Payne effect. An accelerated characterization method is useful for the prediction of the hysteresis loss of rubber products using small amounts of experimental data, which can provide manufacturers with more attractive and lower cost opportunities for testing the mechanical properties of rubber products.

## 1. Introduction

In the building field, viscoelastic dampers are gaining more importance because of the burgeoning demand for the construction of high-rise buildings. It has been reported that the viscoelastic properties of structural components have a significant influence on the reliability of structures, and neglecting to examine the viscoelastic behavior of components can affect the functionality of engineering structures, which can result in structural collapse [1]. Given the increasing number of investigations into, and applications of, viscoelastic dampers, viscoelastic materials such as CB-filled rubber vulcanizate are becoming more commonly used in complex environments [2]. CB-filled rubber vulcanizates have been widely used because of their excellent mechanical properties. As new building materials, they have become commonly used in civil infrastructures, such as building isolators and dampers [3]. In reality, they are usually subject to various preloading conditions in many applications, and their viscous properties play an important role in structural applications [4]. It is worth noting that strain amplitude dependence, which is the so-called Payne effect, might be significant when rubber-like materials are heavily filled [5]. It has been found that the Payne effect is closely linked with the adaptability of filled rubber vulcanizates to changes in load parameters [6]. In addition, the area of the hysteresis loop can reflect the damping capacity of the material [2]. Therefore, it is necessary to understand the mechanism of the Payne effect and hysteresis loss when prestrain is superimposed with small dynamic strain.

CB-filled rubber vulcanizates present obvious nonlinear mechanical properties, which are sensitive to temperature, frequency, prestrain, and amplitude of excitation. Mechanical change can bring irreversible losses to people’s lives and result in severe safety issues. The Payne effect is a nonlinear mechanical property and is quite complex as viscoelasticity takes part in damage development [7]. One of the typical features of CB-filled rubber vulcanizates under the Payne effect is that the dynamic moduli are dependent on the strain amplitude. Under dynamic loading conditions, the storage modulus of CB-filled rubber vulcanizate decreases monotonically, and the loss modulus of CB-filled rubber vulcanizate increases at first, and then decreases with increasing dynamic strain amplitude [8]. To characterize and model the Payne effect, many studies have been conducted from experimental and theoretical angles, under various temperature and frequency conditions [9,10]. Because of practical engineering preloading applications, researchers have shown great interest in the prestrain-dependent Payne effect. Thorin [11] investigated the influence of prestrain on the viscoelastic properties of elastomers using dynamic mechanical analysis (DMA) and observed nonlinear behavior in terms of prestrain. Cho [12] carried out dynamic mechanical tests to investigate the effect of prestrain on dynamic moduli, and the results showed that the storage and loss moduli decreased initially and subsequently increased with increasing prestrain, which was attributed to the limited extensibility of elastomer chains. However, Rendek [13] focused on the Payne effect of filler-reinforced rubber vulcanizates using an experimental investigation and observed an increase in storage and loss moduli with increasing prestrain. Suphadon [14] explained the prestrain dependence of rubber vulcanizates by considering molecular orientation in combination with molecular slippage taking place at the polymer–filler interface. Recently, an increasing number of researchers have used SEM, NMR, and AFM to investigate the Payne effect from the perspective of microstructure evolution [15,16,17]. In addition, hysteresis loss takes place under dynamic loading conditions because of the viscoelasticity of CB-filled rubber vulcanizates. Hysteresis loss can generate heat within CB-filled rubber vulcanizates, which can seriously affect dynamic mechanical properties with increasing temperature [18]. In the engineering field, research shows that vibration damping can be estimated by the amount of energy dissipated during cyclic deformation, which can be calculated from the area of the hysteresis loop [19]. In addition, lack of communication between structural engineers and rubber technologists leads structural engineers to have a lack of understanding regarding rubber properties [4]. Therefore, it is worthwhile to investigate the Payne effect and the hysteresis loss of CB-filled rubber vulcanizates.

Meanwhile, in the process of Payne effect investigations, a series of constitutive models have been proposed. In the classical theoretical framework, the Kraus, Huber–Vilgis, and Maier–Göritz models are the three commonly used models. The most well-known model was proposed by Kraus [20], which was based on filler network breakage and assumed that there was a dynamic equilibrium between the breakage and recovery of weak physical bonds under dynamic loading conditions. Later, Huber and Vilgis [21] developed a model based on the Kraus model, which also attributed the Payne effect to the breakage and reformation of the filler network. However, the commonality of the above two models is that they are either only valid in the frequency domain or for cyclic deformations [22]. In addition, Maier and Göritz [23] proposed a model to describe the Payne effect based on filler–rubber interactions and believed that there were two kinds of filler–rubber bonds: strong bonds and weak bonds. Recently, Lion [24] proposed a modified Kraus model with a time-domain formulation. Based on the theory of nonlinear thermoviscoelasticity, some other models have been proposed to describe the Payne effect. For example, Lion and Kardelky [25] employed a model by means of a series of nonlinear Maxwell elements. Based on an eight-chain representation of the underlying macromolecular network structure of the rubber and the non-Gaussian behavior of individual chains, Arruda and Boyce [26] proposed an eight-chain network model. In addition, for the continuum damage model of stress softening, there are other inelastic models. For example, Ayoub [27] proposed a model integrating the physics of polymer chains and their alteration under dynamic loading conditions, which took stress softening into consideration. However, compared with the above three commonly used models, most of the existing models describing the Payne effect are so complicated that the determination of material parameters is not an easy task [7]. Therefore, in the interest of simplicity, it is meaningful to distinguish the applicability of the three commonly used models.

To this end, this paper attempts to investigate the prestrain-dependent Payne effect and hysteresis loss of CB-filled rubber vulcanizates for potential use in vibration damping. The differences between the present investigation and other studies include the following two main aspects. On the one hand, in the classical theoretical framework, the above three commonly used models are employed to model the prestrain-dependent Payne effect, which can provide guidance for researchers to choose an accurate model for future investigation. On the other hand, by use of the time–strain superposition principle, the hysteresis loss at an arbitrary prestrain can be predicted, which can give manufacturers more attractive and lower cost opportunities for the mechanical properties testing of rubber products.

## 2. Materials and Methods

### 2.1. Hysteresis Loss

**Figure 1:**
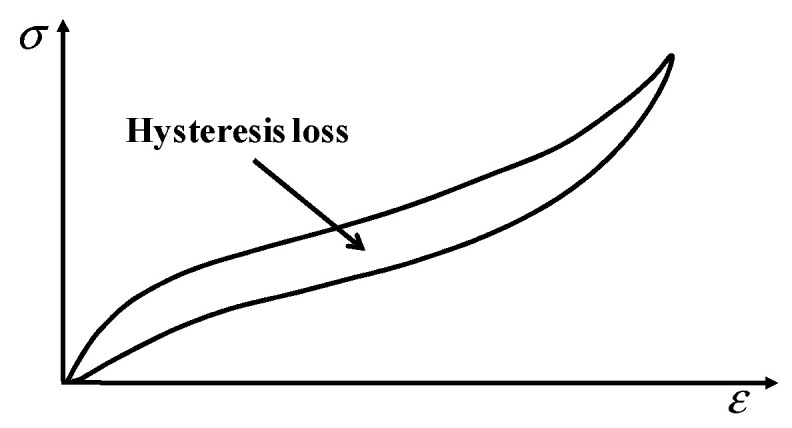
Hysteresis loss calculation for a cyclic loading curve.

Hysteresis loss is defined as the amount of energy dissipated during cyclic deformation when the testing samples are stretched and then retract to the unstretched state at the same rate [28]. Under dynamic mechanical test, the stress–strain curve can be recorded, and the hysteresis loss *D* shown in Figure 1 can be calculated as

*D* = A_loading_ − A_recovery_ (1)

where A_loading_ is the area under the loading curve, and A_recovery_ is the area under the recovery curve.

To investigate the prestrain-dependent Payne effect and hysteresis loss, the test specimen is loaded with a large prestrain $\varepsilon_{0}$ and a superimposed harmonic excitation of small amplitude $\varepsilon_{\Delta }$. The strain excitation *ε*(*t*) can be expressed as

(2) $$\varepsilon \left(t\right)=\varepsilon_{0}+\varepsilon_{\Delta }\sin\left(\omega t\right)$$

where ω is the angular frequency. Within certain strain amplitude limits, it is a common practice to neglect higher-order terms in the material response, and the stress response can be written as [22]

(3) $$\sigma \left(t\right)=\sigma_{0}+\varepsilon_{\Delta }\left[E^{\prime }\sin\left(\omega t\right)+E^{\prime \prime }\cos\left(\omega t\right)\right]$$

where $E^{\prime }$ and $E^{\prime \prime }$ are the storage modulus and the loss modulus, respectively. And $\sigma_{0}$ represents static stress, which is a function of prestrain.

Under cyclic loading, the calculation of strain energy *W* over one period can be expressed by the following equation:

(4) $$W=\int_{0}^{\frac{2\pi }{\omega }}\sigma d\varepsilon =\omega {\left(\varepsilon_{\Delta }\right)}^{2}\int_{0}^{\frac{2\pi }{\omega }}\left[E^{\prime }\sin\left(\omega t\right)\cos\left(\omega t\right)+E^{\prime \prime }\cos^{2}\left(\omega t\right)\right]dt$$

Under cyclic loading, the energy includes stored energy and dissipated energy. The dissipated energy, named hysteresis loss, can be calculated by the area of hysteresis loop, which can generate heat within the CB-filled rubber vulcanizates and can seriously affect the dynamic mechanical properties with increasing temperature [18]. In addition, it is worth noting that only the loss modulus is involved in the dissipated energy. Therefore, Equation (1) can be rewritten as

(5) $$D=\pi {\left(\varepsilon_{\Delta }\right)}^{2}E^{\prime \prime }$$

### 2.2. Models

#### 2.2.1. Kraus Model

To investigate the amplitude-dependent Payne effect, the most well-known model is proposed by Kraus, which is the so-called Kraus model. It is based on filler network breakage, which supposes a dynamic equilibrium of weak physical bonds in the processes of breakage and recovery. In this model, the amount of network breakage per cycle *R*_b_ is proportional to the number of existing CB contacts *N* as well as a function of strain amplitude *f*_b_:

(6) $$R_{b}=k_{b}Nf_{b}$$

where *k*_b_ is a broken rate constant. In addition, the network reformation rate *R*_r_ is proportional to the number of broken contacts as well as a function of strain amplitude *f*_r_:

(7) $$R_{r}=k_{r}\left(N_{0}-N\right)f_{r}$$

where *k*_r_ is a constant, and *N*_0_ is the number of intact contacts in the network. At dynamic equilibrium, *R*_b_*= R*_r_. Because of the power law relationships of *f*_b_ and *f*_r_ with the dynamic amplitude Δε: $f_{b}={\left(\varepsilon_{\Delta }\right)}^{m}$, $f_{r}={\left(\varepsilon_{\Delta }\right)}^{-m}$, taking Equations (6) and (7) into consideration, the Kraus model for storage modulus $E^{\prime }$ can be derived as [8,29]

(8) $$E^{\prime }\left(\varepsilon_{\Delta }\right)=E_{\prime }^{\infty }+\frac{E_{0}^{\prime }-E_{\infty }^{\prime }}{1+{\left(\varepsilon_{\Delta }/\varepsilon_{\Delta ,c}\right)}^{2m}}=E_{\prime }^{0}-\Delta E^{\prime }+\frac{\Delta E^{\prime }}{1+{\left(\varepsilon_{\Delta }/\varepsilon_{\Delta ,c}\right)}^{2m}}$$

where $\varepsilon_{\Delta ,c}$ is a characteristic value of strain amplitude; $E_{0}^{\prime }$ is the storage modulus when dynamic strain amplitude is less than 0.01%; $E_{\infty }^{\prime }$ is an asymptotic plateau value of the storage modulus at large strain amplitudes; $\Delta E^{\prime }\left(=E_{0}^{\prime }-E_{\infty }^{\prime }\right)$ is the excess storage modulus; and *m* is a nonnegative phenomenological exponent.

According to Kraus, a loss modulus $E^{\prime \prime }$ in excess of the loss modulus at infinite strain $E_{\infty }^{\prime \prime }$ arises from the energy dissipation associated with the breakage and recovery of individual contact. In other words:

(9) $$E^{\prime \prime }\left(\varepsilon_{\Delta }\right)-E_{\infty }^{\prime \prime }=C_{1}k_{b}Nf_{b}$$

where *C*_1_ is a constant. With $f_{b}={\left(\varepsilon_{\Delta }\right)}^{m}$, Equation (9) can be differentiated to obtain an expression for the maximum loss modulus $E_{m}^{\prime \prime }$, which can be used to derive the loss modulus expression of the Kraus model [8,29]:

(10) $$E^{\prime \prime }\left(\varepsilon_{\Delta }\right)=E_{\infty }^{\prime \prime }+\frac{2\left(E_{m}^{\prime \prime }-E_{\infty }^{\prime \prime }\right){\left(\varepsilon_{\Delta }/\varepsilon_{\Delta ,c}\right)}^{m}}{1+{\left(\varepsilon_{\Delta }/\varepsilon_{\Delta ,c}\right)}^{2m}}$$

where $E_{m}^{\prime \prime }$ is the maximum value of the loss modulus when $\varepsilon_{\Delta }=\varepsilon_{\Delta ,c}$ and $E_{\infty }^{\prime \prime }$ is an asymptotic plateau value of the loss modulus at large strain amplitudes.

#### 2.2.2. Huber–Vilgis Model

It should be noted that both the Huber–Vilgis model and Kraus model attribute the Payne effect to the breakage and reformation of the filler network. Because of their similarity, the introduction of the Huber–Vilgis model will not be discussed in detail in this section. In the Huber–Vilgis model, the storage and loss moduli are expressed by the following two equations [29]:

(11) $$\frac{E^{\prime }\left(\varepsilon_{\Delta }\right)-E_{\infty }^{\prime }}{E_{0}^{\prime }-E_{\infty }^{\prime }}=\frac{1}{1+K^{2}{\left(\varepsilon_{\Delta }\right)}^{2m}}$$

(12) $$\frac{E^{\prime \prime }\left(\varepsilon_{\Delta }\right)-E_{\infty }^{\prime \prime }}{E_{0}^{\prime }-E_{\infty }^{\prime }}=\frac{2\alpha K{\left(\varepsilon_{\Delta }\right)}^{m}}{1+K^{2}{\left(\varepsilon_{\Delta }\right)}^{2m}}$$

Letting $K={\left(1/\varepsilon_{\Delta ,c}\right)}^{m}$ and $E_{m}^{\prime \prime }-E_{\infty }^{\prime \prime }=\alpha \left(E_{0}^{\prime }-E_{\infty }^{\prime }\right)$, it can be seen that the Huber–Vilgis model is equivalent to the Kraus model. Therefore, the results of the experimental data analysis by the Huber–Vilgis model are not presented in this paper.

#### 2.2.3. Maier–Göritz Model

As well as the Kraus model and Huber–Vilgis model, the Maier–Göritz model has often been used to describe the Payne effect, which is based on filler–rubber interactions. In this model, it is believed that there are two kinds of filler–rubber bonds in CB-filled rubber vulcanizates: strong bonds and weak bonds [30]. During mixing, rubber chains are adsorbed onto filler, first forming strong/stable bonds. After the formation of stable bonds on the surface of the filler, there is less opportunity for the remaining chain to contact and bond with the filler, which results in the formation of weak/unstable bonds. In addition, in a filled rubber compound, the bonds contribute to network density, which is directly related to the dynamic moduli and Payne effect [31]. In the Maier–Göritz model, the storage and loss moduli versus strain are given by the following two equations [29]:

(13) $$E^{\prime }\left(\varepsilon_{\Delta }\right)=E_{\mathrm{st}}^{\prime }+E_{i}^{\prime }\frac{1}{1+c\varepsilon_{\Delta }}$$

(14) $$E^{\prime \prime }\left(\varepsilon_{\Delta }\right)=E_{\mathrm{st}}^{\prime \prime }+E_{i}^{\prime \prime }\frac{c\varepsilon_{\Delta }}{{\left(1+c\varepsilon_{\Delta }\right)}^{2}}$$

where $E_{\mathrm{st}}^{\prime }$ is the storage modulus at large deformation amplitudes or strain; $E_{i}^{\prime }$ gives the Payne effect amplitude; *c* is a parameter determined by experiments, which reflects the position of the maximum value of the loss modulus on the dynamic strain axis; $E_{\mathrm{st}}^{\prime \prime }$ represents the loss modulus when dynamic strain amplitude has a very high or low value; and $E_{i}^{\prime \prime }$ represents the variation amplitude of the loss modulus.

### 2.3. Materials

The CB-filled natural rubber material used for dynamic mechanical testing with Shore-A hardness of 66 was provided by Zhuzhou Times New Material Technology Co., Ltd., Zhuzhou, China. The main formulation of the rubber compound is as follows: 100 phr natural rubber (RRS3, provided by Taihua Group, Bangkok, Thailand), 2.2 phr sulphur, and 0.8 phr vulcanization accelerator. Further details on the content of CB (N330) cannot be given for reasons of privacy. The specimens were thin rectangular strips with dimensions of 25 × 5 × 2 mm^3^.

### 2.4. Dynamic Mechanical Analysis

To investigate the prestrain-dependent properties of CB-filled rubber material, dynamic strain amplitude sweep tests were carried out in tensile mode with a Gabo Eplexor 500N, NETZSCH-Gerätebau GmbH, Selb, Germany. Before the dynamic mechanical tests, Mullins effect should be excluded. And the specimens were preconditioned with a cyclic strain-controlled process. In this process, the first 6 cycles were selected to exclude the Mullins effect. And it is worth noting that the dynamic strain amplitude was not less than the maximum strain amplitude in the subsequent Payne effect investigation.

It is generally recognized that the Payne effect represents a softening process of the elastic modulus of rubber products, which can be observed in dynamic mechanical tests for strain values typically between 0.1% and 10% [32]. Therefore, in this work, the specimens were sinusoidally stretched with prestrains, i.e., 1%, 2%, 3%, 4%, 5%, and 6% at room temperature. The loading frequency was 10 Hz, and the dynamic strain amplitudes ranged from 0.1% to 1% with steps of 0.05%. In the process of testing, the dynamic moduli were recorded, and the hysteresis loops in full cycles were constructed.

## 3. Results

### 3.1. Payne Effect

**Figure 2:**
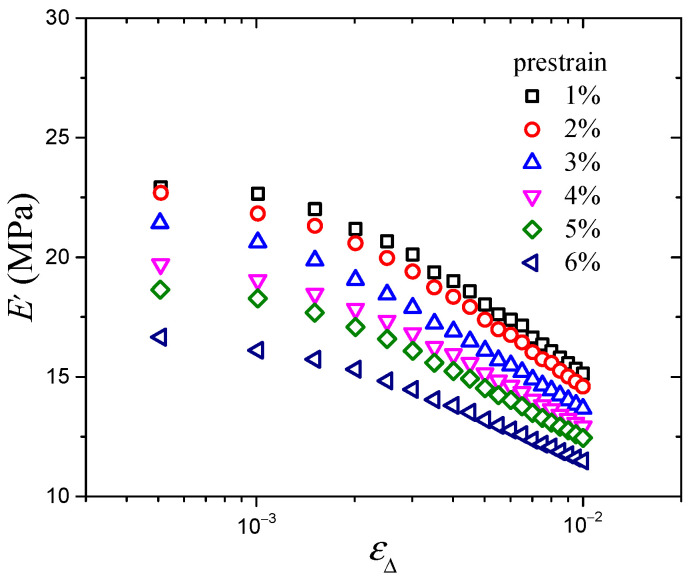
Storage modulus vs. strain amplitude at various prestrains.

**Figure 3:**
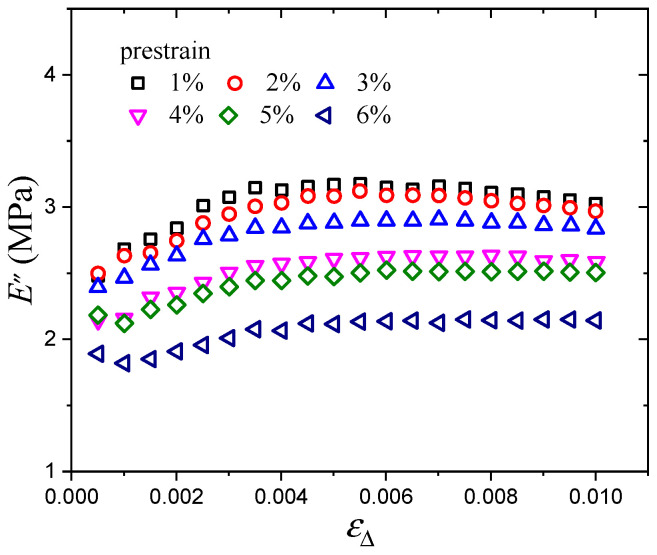
Loss modulus vs. strain amplitude at various prestrains.

The prestrain-dependent dynamic mechanical testing results of the CB-filled rubber vulcanizates are shown in Figure 2 and Figure 3. With increasing dynamic strain amplitude, the storage modulus decreases monotonically, and the loss modulus increases at first and then decreases. In all cases, both the storage modulus and loss modulus decrease with increasing prestrain, which is mainly due to the decrease in the network chain density of the material during the dynamic loading process when the stretch ratio increases [33]. And in the process of dynamic deformation, Du [34] pointed out that the prestrain-dependent behavior is mainly because of the fact that the existence of prestrain will destroy the micromolecular chain structure and reduce the crosslinking density of the material. Moreover, Cho [12] found that the storage and loss moduli of CB-filled rubber decreased initially and subsequently increased with increasing prestrain, and the critical prestrain amplitude of the storage and loss moduli trend change was about 10%, which verified the reliability of our experimental results.

In the process of investigating the Payne effect, a series of constitutive models have been proposed to describe this kind of mechanical behavior. As mentioned in Section 1, compared with the three commonly used models, most of the other existing models describing the Payne effect are so complicated that the determination of material parameters is not an easy task [7]. Therefore, in the interest of simplicity, the Kraus model, Huber–Vilgis model, and Maier–Göritz model are used to model the Payne effect, and it is meaningful to distinguish the applicability of the three commonly used models. In addition, the Huber–Vilgis model is not considered in this paper because of the similarity between the Huber–Vilgis model and Kraus model.

**Figure 4:**
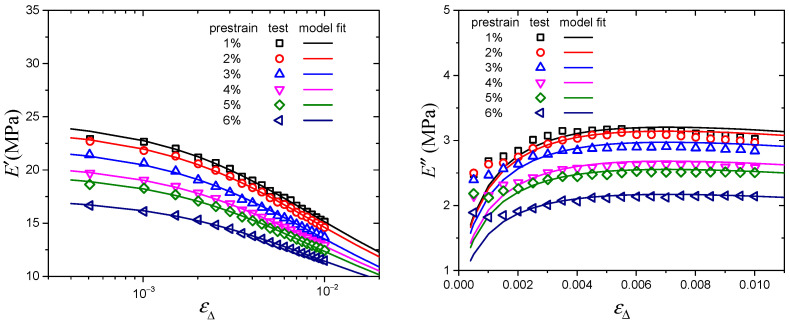
Experimental data and the Kraus model fitting results.

**Figure 5:**
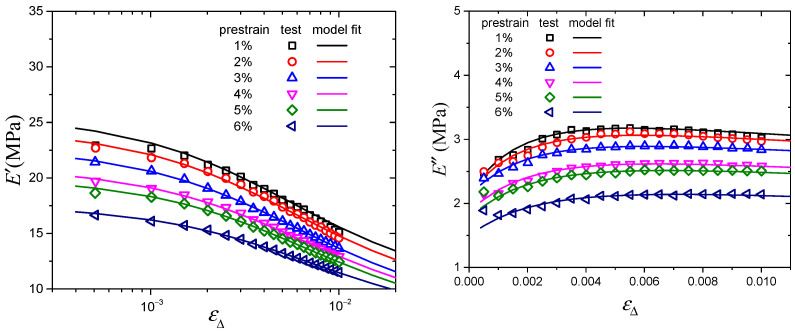
Experimental data and the Maier–Göritz model fitting results.

Figure 4 and Figure 5 show the modeling results of the Kraus model and Maier–Göritz model. From the modeling results, it is obvious that both the Kraus model and Maier–Göritz model can describe the storage modulus well. With regard to the loss modulus in Figure 4, it can be seen that the Kraus model is not able to describe the dissymmetric shape experimentally observed. Moreover, in the process of modeling the Payne effect, the limitations of the Kraus model have also been identified. For example, it is reported that the Kraus model does not take temperature effect into consideration [29]. As for the Huber–Vilgis model, because of the similarity between this model and the Kraus model, it has the same limitations as the Kraus model in describing the Payne effect. However, for the loss modulus, the Maier–Göritz model has a better fitting effect, especially when dynamic strain amplitude is less than 0.2%. Therefore, it can be concluded that the Maier–Göritz model is more practical to use to model the prestrain-dependent Payne effect, which can provide guidance for researchers to choose an accurate model for future investigation.

**Figure 6:**
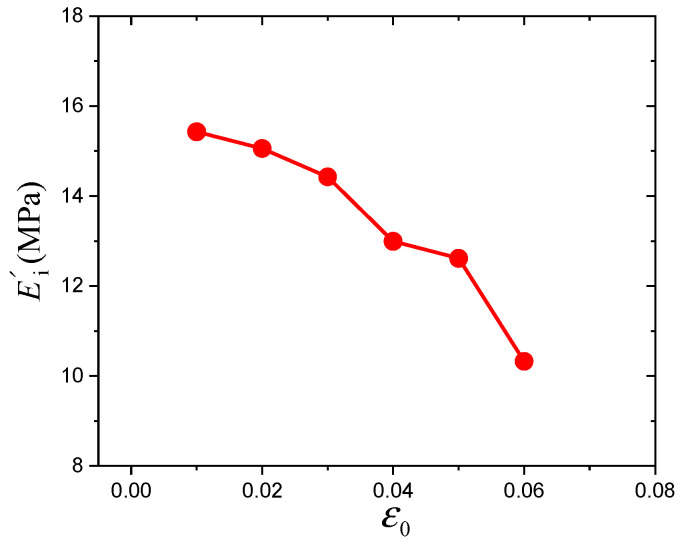
Variation of parameter $E_{i}^{\prime }$ with prestrain.

**Figure 7:**
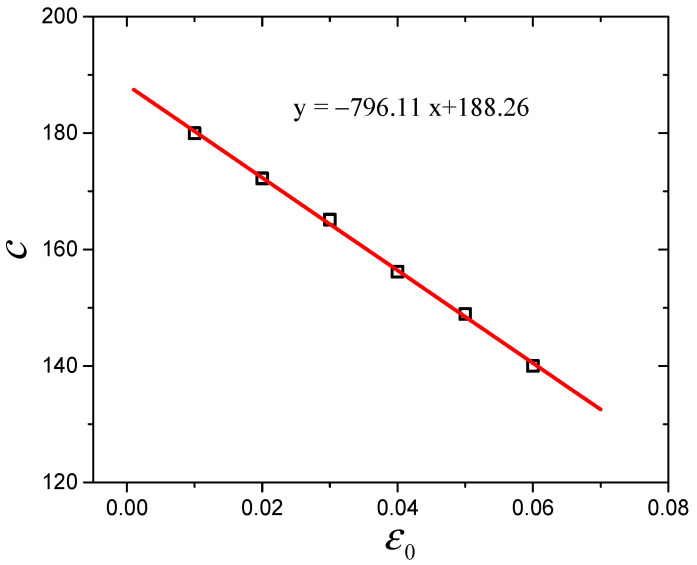
Variation of parameter *c* with prestrain.

Here, the parameters of Equations (13) and (14) are presented in Table 1 and will be discussed in detail. *R* presents the fitting correlation coefficient. As shown in Table 1, all parameters of the Maier–Göritz model decrease gradually with increasing prestrain. In the Maier–Göritz model, $E_{i}^{\prime }$ is the amplitude of the Payne effect, as shown in Figure 6. There is a decreasing trend, which is mainly because the existence of prestrain will destroy the micromolecular chain structure and reduce the crosslinking density of the material [34]. Parameter *c* defines the position of the maximum value of the loss modulus along the dynamic strain axis, and the relationship between parameter *c* and the prestrain has been established, as shown in Figure 7. It is observed that they are linearly related. $E_{i}^{\prime \prime }$ gives the variation amplitude of the loss modulus, which relates to the hysteresis loss and will be discussed in the following section.

### 3.2. Hysteresis Loss

**Figure 8:**
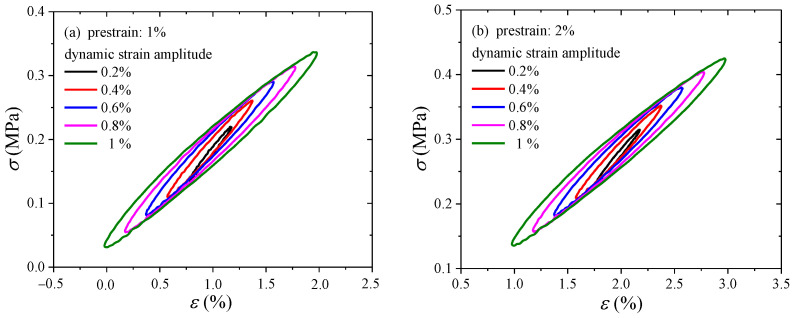

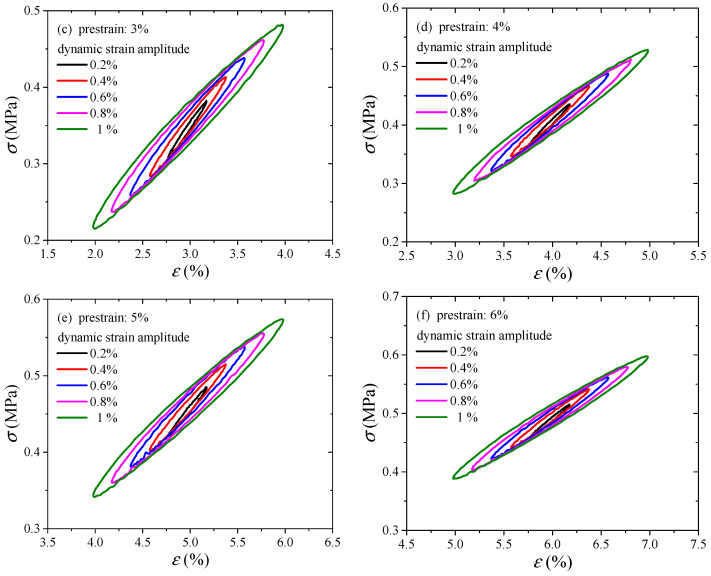
Hysteresis loops for various prestrains: (**a**) 1%; (**b**) 2%; (**c**) 3%; (**d**) 4%; (**e**) 5%; (**f**) 6%.

The hysteresis effect can affect the building isolator performance characteristics. For engineering applications, hysteretic analysis is performed in order to determine the damping properties of isolators, such as the damping ratio [35]. Therefore, it is meaningful to investigate the hysteresis loss. Under cyclic loading, in the process of the Payne effect test, the CB-filled rubber vulcanizates exhibit hysteresis loss because of the phase lag between stress and strain. Since there are many hysteresis loops and there is no need to show all the loops, the following hysteresis loops are chosen as examples under various dynamic strain amplitudes, i.e., 0.2%, 0.4%, 0.6%, 0.8%, and 1%. The hysteresis loops in Figure 8 are obtained when plotting steady stress versus strain for harmonic excitation. As observed in Figure 8, the hysteresis loops at all strain amplitudes are nearly elliptic.

**Figure 9:**
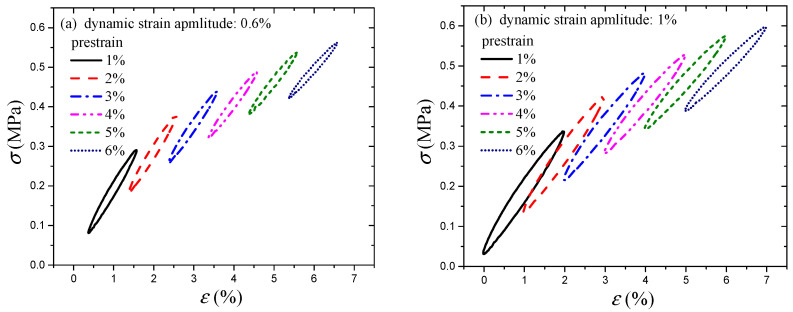
Hysteresis loops at various prestrains and two given strain amplitudes: (**a**) 0.6%; (**b**) 1%.

**Figure 10:**
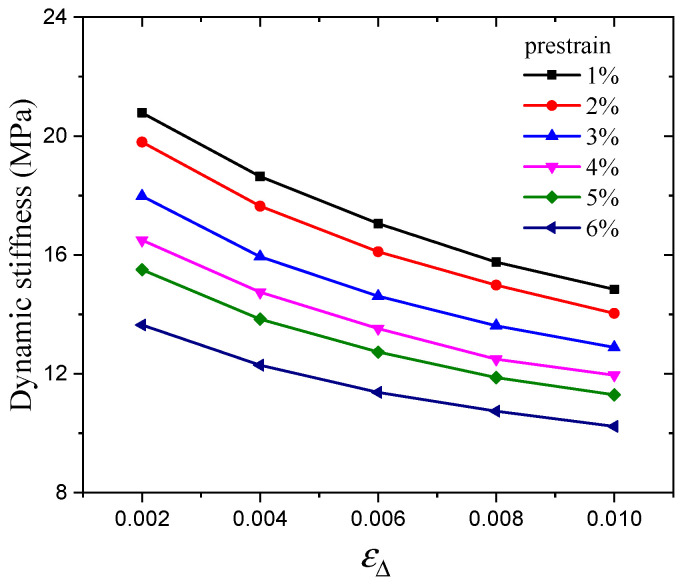
Relationship between stiffness and dynamic strain amplitudes under various prestrains.

**Figure 11:**
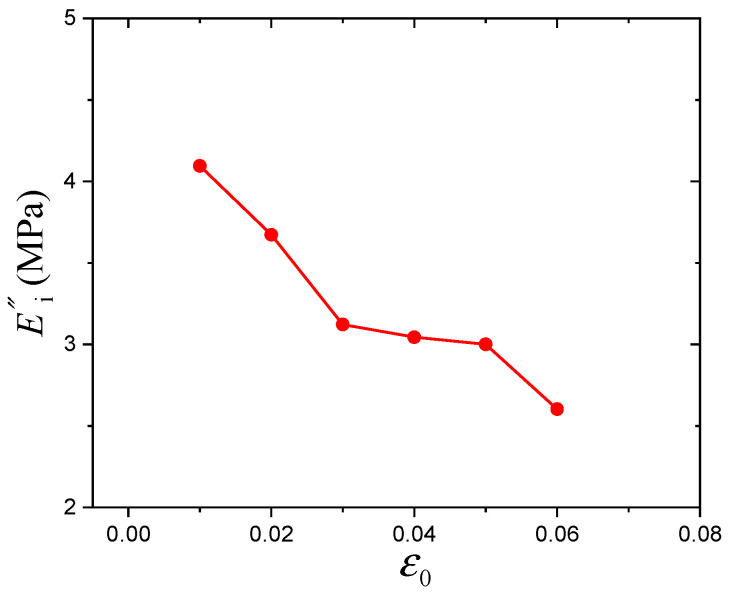
Variation of parameter $E_{i}^{\prime \prime }$ with prestrain.

In addition, the effect of prestrain on the hysteresis loss of CB-filled rubber vulcanizates under cyclic loading will be examined and assessed. The stiffness is related to the slope of the hysteresis loop (measured from the maximum point to the minimum point). Because of the similarity, hysteresis loops under dynamic strain amplitudes of 0.6% and 1% are taken as examples, as shown in Figure 9. It is obvious that the slopes decrease with increasing prestrain. In addition, the slope calculation results of the hysteresis loops are shown in Figure 10. This behavior can reflect the Payne effect. The results show that the slope/stiffness of the hysteresis loops slightly decreases nonlinearly with increasing dynamic strain amplitude, which can result in a decrease in loss modulus [36]. Figure 10 shows that the stiffness also has the same trend with increasing prestrain. Notably, the relationship between the stiffness and loss modulus can be verified by the Payne effect modeling results. In the Maier–Göritz model, parameter $E_{i}^{\prime \prime }$ gives the variation amplitude of the loss modulus, as shown in Figure 11. Figure 11 indicates that the loss modulus decreases with increasing prestrain, which can be verified by the testing results in Figure 3.

**Figure 12:**
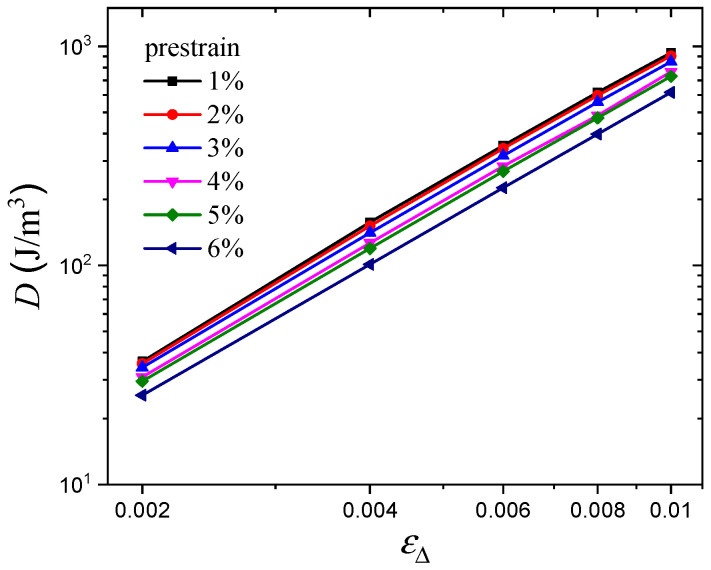
Hysteresis loss for various prestrains.

**Figure 13:**
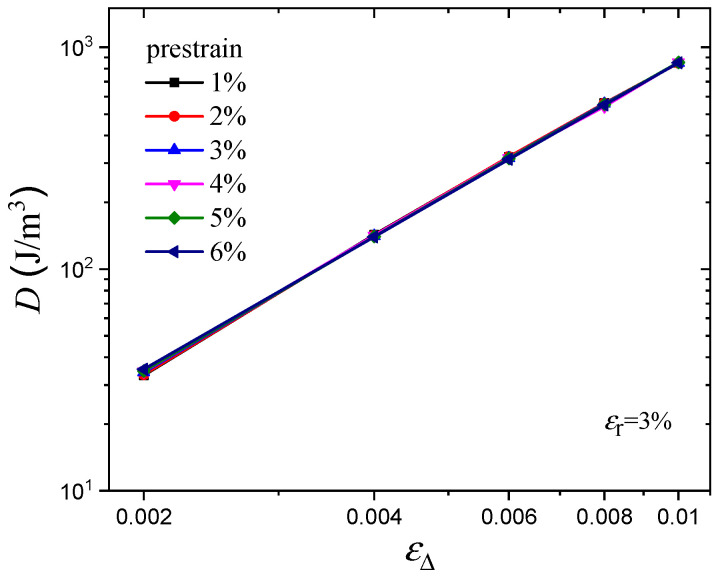
Superimposed curve of hysteresis loss.

In addition, the energy dissipation can be quantified by the area of the hysteresis loop, as shown in Figure 12. The results show that, in the double logarithmic coordinate, there is a linear relationship between hysteresis loss *D* and dynamic strain amplitude $\varepsilon_{\Delta }$, which indicates that there is a power relationship for the *D*~$\varepsilon_{\Delta }$ curve in the linear coordinate. Moreover, it is apparent that the hysteresis loss curves under various prestrains are parallel to each other, which indicates that the hysteresis loss at any arbitrary prestrain can be predicted by the use of vertical shift factors. It is well known that shift factor determination is a very useful method for accelerated characterization of mechanical properties. Therefore, a superimposed curve at the reference prestrain of 3% was constructed, as shown in Figure 13, and the shift relationship can be expressed by

(15) $$D\left(\varepsilon_{\Delta },\varepsilon_{0}\right)=\phi \cdot D\left(\varepsilon_{\Delta },\varepsilon_{0,r}\right)=\phi \cdot q\cdot {\left(\varepsilon_{\Delta }\right)}^{k}$$

where *D* is the hysteresis loss, ϕ is the vertical shift factor, $\varepsilon_{0,r}$ is the reference prestrain, and *k* is the exponent of the power function, which is set to be a constant because the log*D*~log$\varepsilon_{\Delta }$ curves are parallel to each other. The shift factors and the relationship between *D* and $\varepsilon_{\Delta }$ are shown in Table 2.

**Figure 14:**
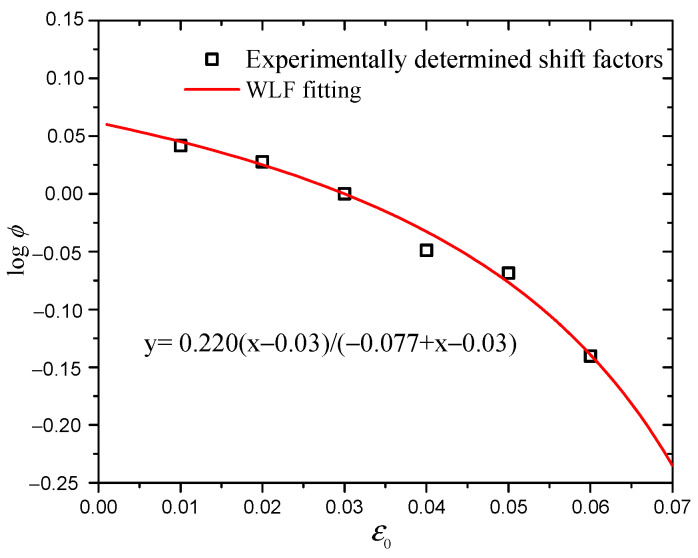
Experimentally determined shift factors vs. prestrain, fit to the WLF equation.

It is well known that the dynamic mechanical behaviors of CB-filled rubber vulcanizates are strongly temperature-dependent. The time–temperature superposition principle (TTSP) can be employed to describe the relationship between time and temperature. To obtain the long-term mechanical properties of elastomers, the WLF equation has been widely used in the temperature range *T* > *T*_g_. Similarly, for strained cases, there is a time–strain superposition principle, and the time–strain shift factor can also be rewritten as another WLF equation [37], as shown in Equation (16). In the process of constructing the superimposed curve at a reference prestrain of 3%, the vertical shift factors are obtained and fitted by the WLF equation, as shown in Figure 14. The results show that the vertical shift factors satisfy the WLF equation very well, which indicates that the hysteresis loss at an arbitrary prestrain can be predicted by use of the WLF equation.

(16) $$\log\phi =-C_{1}\frac{\left(\varepsilon_{0}-\varepsilon_{0,r}\right)}{C_{3}+\left(\varepsilon_{0}-\varepsilon_{0,r}\right)}$$

where *C*_1_ and *C*_3_ are the material constants.

In this section, the prestrain-dependent Payne effect and hysteresis loss of CB-filled rubber vulcanizates have been comprehensively investigated through theoretical study and experimental analysis. For engineering applications, the rolling resistance of a tire is closely related to the Payne effect of the material [38]; thus, this paper can provide manufacturers with a more attractive and lower cost approach to the production of fuel-efficient rubber products with improved performance characteristics. Moreover, hysteretic analysis can be used to determine the damping properties of the isolator, such as the damping ratio [35]. Therefore, under dynamic deformation, the accelerated characterization method is useful to predict the damping properties of rubber products by use of small amounts of experimental data.

## 4. Conclusions

In this paper, the prestrain-dependent Payne effect and hysteresis loss were experimentally investigated, and three classical models were employed to model the Payne effect. The results show that the Maier–Göritz model is more suitable to describe the Payne effect, especially for the loss modulus, which provides guidance for researchers to choose an accurate model for the future investigation of the prestrain-dependent Payne effect. In addition, the relationships between the parameters in the Maier–Göritz model and the prestrain were determined. For hysteresis loss, it is apparent that the hysteresis loss curves under various prestrains are parallel to each other. Therefore, hysteresis loss at an arbitrary prestrain can be predicted by use of vertical shift factors.

## Figures and Tables

**Table 1:** Parameter values of the Maier–Göritz model.

$\varepsilon_{0}/\%$	$E_{\mathrm{st}}^{\prime }$/MPa	$E_{i}^{\prime }$/MPa	$E_{\mathrm{st}}^{\prime \prime }$/MPa	$E_{i}^{\prime \prime }$/MPa	*c*	*R*
1	10.083	15.429	2.151	4.095	179.96	0.999
2	9.255	15.057	2.146	3.672	172.20	0.983
3	8.219	14.425	2.110	3.122	165.12	0.993
4	7.877	12.995	1.856	3.044	156.18	0.990
5	7.351	12.616	1.764	3.001	148.90	0.996
6	7.169	10.325	1.490	2.603	140.01	0.991

**Table 2:** Shift factors and relationship between *D* and $\varepsilon_{\Delta }$.

$\varepsilon_{0}/\%$	$D=q{\left(\varepsilon_{\Delta }\right)}^{k}$	ϕ
1	$9,456,324{\left(\varepsilon_{\Delta }\right)}^{2.0}$	1.1009
2	$9,240,044{\left(\varepsilon_{\Delta }\right)}^{2.0}$	1.0656
3	$8,671,529{\left(\varepsilon_{\Delta }\right)}^{2.0}$	1.0000
4	$7,748,304{\left(\varepsilon_{\Delta }\right)}^{2.0}$	0.8935
5	$7,403,709{\left(\varepsilon_{\Delta }\right)}^{2.0}$	0.8537
6	$6,276,448{\left(\varepsilon_{\Delta }\right)}^{2.0}$	0.7238

## Data Availability

The raw data supporting the conclusions of this article will be made available by the authors on request.
